# Total laparoscopic management of large complicated jejunal diverticulum

**DOI:** 10.4103/0972-9941.59311

**Published:** 2009

**Authors:** Niraj Garg, Rajesh Khullar, Anil Sharma, Vandana Soni, Manish Baijal, Pradeep Chowbey

**Affiliations:** Department of Minimal Access, Metabolic and Bariatric Surgery, Sir Ganga Ram Hospital, New Delhi - 110 060, India

**Keywords:** Diagnostic laparoscopy, diverticulum, resection anastomosis

## Abstract

Jejunoileal diverticulae, also referred to as non-Meckelian diverticulae, are very uncommon. These diverticulae are considered to be acquired pulsion diverticulae and they mostly occur in older people. Their prevalence increases with age. About 80% of these diverticulae occur in jejunum and are usually multiple. Patients with jejunoileal diverticulae present with nonspecific symptoms. The clinical picture of a complicated jejunoileal diverticulae can be confused with other intra-abdominal acute conditions such as appendicitis, cholecystitis, perforated ulcer, etc. Nonmechanical or pseudoobstruction is related to the dyskinesia associated with this condition. The diagnosis is made by a small bowel contrast study, enteroclysis, endoscopy or computed tomography. A surgical approach is the best form of treatment for complicated jejunoileal diverticulae. Laparoscopy is very useful in diagnosing and treating this condition. The current report is about a patient who presented with recurrent subacute intestinal obstruction and was managed by laparoscopy.

## INTRODUCTION

The incidence of jejunoileal diverticulae in the general population on small bowel contrast radiographic series is 0.02-1.3% of the adult population.[1] The clinical presentations of acquired jejunoileal diverticulae are vague and diverse. The complications of jejunoileal diverticulae such as chronic abdominal pain, malabsorption, haemorrhage, diverticulitis, obstruction, abscess in the mesentery and perforation, in occur 10-30% of patients[2] and signal those patients who require operative management. Jejunoileal diverticulitis is quite uncommon and has a mortality rate as high as 24%.[3] We report a patient with complicated jejunal diverticulum managed entirely laparoscopically. This is the first such case of a large jejunal diverticulum managed totally by laparoscopy to be reported in the literature.

## CASE REPORT

**Figure 1:**
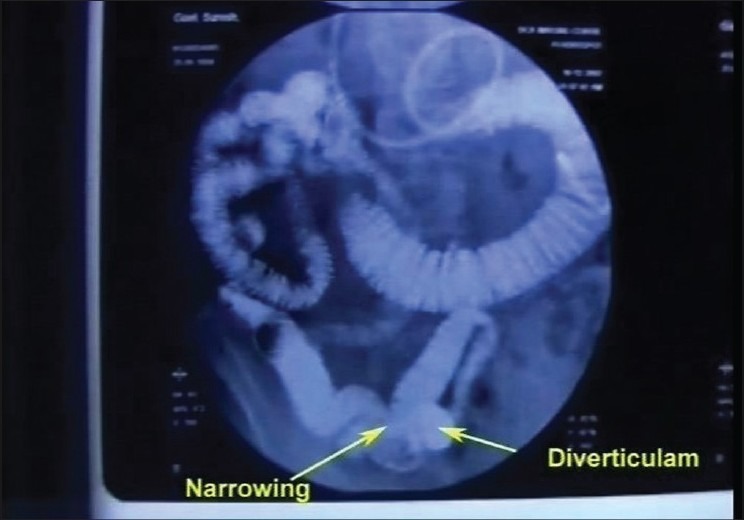
Small bowel enema showing diverticulum

**Figure 2:**
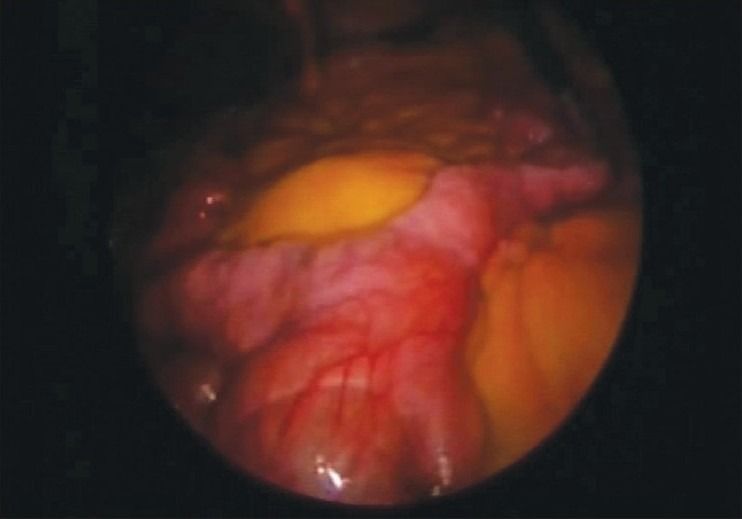
Intraoperative view of diverticulum

A 57-year-old man presented with recurrent episodes of pain in the umbilical region, inability to pass flatus and occasional abdominal distension for 3 months. His abdomen was soft, nontender, and no lump could be felt. A small bowel enema with a tube revealed short segment stricture in the proximal jejunal loop with a small diverticulum proximal to and a large diverticulum beyond this segment [Figure 1]. His double-balloon enteroscopy revealed evidence of kinking of jejunal loop at 40-50 cm from the DJ flexure [Figure 2], causing lumen compromise. There was evidence of large diverticulum seen just distal to the site.

Diagnostic laparoscopy revealed a large (∼8 cm) jejunal diverticulum with proximal dilated loop approximately 50 cm from the DJ flexure. A laparoscopic resection of the jejunal diverticulum with 5 cm bowel margins on both sides was performed with endoscopic linear cutter 45 mm (Ethicon Endosurgery Inc., Cincinnati, OH, USA). Enterotomies were made on both proximal and distal bowel loops on the anti-mesenteric border, and a side-to-side jejuno jejunostomy was performed with an endostapler. The common enterotomy site was closed with intracorporeal sutures. The mesenteric defect was closed with a silk suture. The patient resumed a liquid diet on Day 1 of surgery. A soft diet was started on Day 2. The patient passed stools on postoperative Day 3 and was discharged. The histopathology report revealed a jejunal diverticulum (specimen consists of a segment of intestine with 8 cm diverticulum, serosa and mucosa congested and some degree of crypt hyperplastic villous atrophy in mucosa). At a 6-month follow-up, the patient was asymptomatic.

## DISCUSSION

Acquired jejunoileal diverticulum was first described in 1794 by Sommering and later in 1807 by Sir Astley Cooper and was characterized by herniation of mucosa and submucosa through the muscular layer of the wall on the mesenteric border of the bowel.

Vague and chronic abdominal pain of varying severity, localized epigastrically or periumbilically, with a bloating sensation after food intake is frequent and may be the earliest symptom. Intestinal obstruction secondary to jejunoileal diverticulosis is typically cited as a less common complication, with an estimated incidence of 5%.

Mechanical obstruction may result due to pressure on the intestinal wall from an inflammatory mass or scarring and stenosis of the intestinal lining associated with diverticulitis, intussusception and adhesions. Classically, the diagnosis of small bowel diverticulae is made with radiography such as small bowel contrast series or by an enteroclysis study. Current consensus is that enteroclysis is the most accurate.[4] As a result, the diagnosis is by exclusion of one or the other factors and seldom made before diagnostic laparoscopy or exploratomy laparotomy.[5] Diagnostic laparoscopy is very useful in evaluating patients with a complicated course. It ensures an accurate diagnosis and avoids the risk of unnecessary laparotomy if not indicated.[5]

A more aggressive surgical approach can be justified in treating jejunoileal diverticulosis even in patients with chronic symptoms; hence, the potential benefit of resecting symptomatic multiple jejunoileal diverticulae outweighs the small risk of morbidity after elective small bowel resection.[2]

There are various surgical approaches for diagnosing and treating jejunal diverticulae, such as exploratory laparotomy, diagnostic laparoscopy and conversion to open laparotomy, resection and anastomosis of the diseased small bowel segment; diagnostic laparoscopy, laparoscopic resection and anastomosis of the diseased small bowel segment. Laparoscopic anastomosis can be done by either a suturing technique or by a stapler technique.

## CONCLUSION

Jejunoileal diverticulosis is a rare condition that continues to present formidable challenges in diagnosis and treatment. It should not be regarded as an insignificant finding and should be kept high in the differentials in older patients presenting with unexplained abdominal symptoms because it may lead to life-threatening complication and death. In the presence of complications, surgical resection with primary anastomosis is the preferred treatment option.
